# Protocol for the systematic review of return-to-activity criteria in adolescent patients following an anterior cruciate ligament reconstruction

**DOI:** 10.1186/s13643-022-01965-w

**Published:** 2022-05-14

**Authors:** Nicholas J. Romanchuk, Holly Livock, Kenneth J. Lukas, Michael J. Del Bel, Daniel L. Benoit, Sasha Carsen

**Affiliations:** 1 Ottawa Carleton Institute for Biomedical Engineering, University of Ottawa, 800 King Edward Ave., Ottawa, ON K1N 6N5 Canada; 2 Division of Orthopaedic Surgery, Children’s Hospital of Eastern Ontario Research Institute, 401 Smyth Rd, Ottawa, K1H 8L1 Canada; 3 Trinity College, University of Dublin, College Green, Dublin 2, Dublin, Ireland; 4 School of Rehabilitation Sciences, University of Ottawa, 451 Smyth Road, Ottawa, ON K1H 8M5 Canada; 5 School of Human Kinetics, University of Ottawa, 125 University, Ottawa, ON K1N 6N5 Canada

**Keywords:** Anterior cruciate ligament, Anterior cruciate ligament reconstruction, Return to activity, Functional tests, Pediatric, Adolescent

## Abstract

**Background:**

Anterior cruciate ligament (ACL) rupture is a debilitating knee injury associated with sequela such as joint instability and progressive degeneration. Unfortunately, following surgical ACL reconstruction in adolescents, the rates of ACL graft failure range from 17 to 19%. A contributing factor to the high reinjury rate in this population may be the limited evidence regarding appropriate criteria for allowing unrestricted return-to-activities (RTA) postoperatively. Several systematic reviews have already sought to develop a consensus on what criteria should be utilized for releasing patients to unrestricted sports activities; however, these reviews have focused on adult populations, a group at much lower risk for reinjury. Our objective is to systematically examine the literature and identify the criteria used when determining unrestricted RTA following an ACL reconstruction in an adolescent population.

**Methods:**

Following the Preferred Reporting Items for Systematic Reviews and Meta-Analyses guidelines, a systematic search will be performed of the MEDLINE/PubMed, Cochrane, Embase, CINAHL, and SPORTDiscus electronic databases. Searches will be conducted from January 1, 2000, until submission of the final review. Studies will be identified that include adolescent patients (10–18 years old) undergoing a primary ACL reconstruction and which have specified the criteria used to determine RTA. Each article will be independently screened by two reviewers. To supplement the electronic database search, citations within all included studies will be manually reviewed. Reviewers will record the RTA assessment utilized and the rates of ACL reinjury through a standardized data extraction sheet. Reviewers will resolve full-text screening and data extraction disagreements through discussion. Synthesis of the collected data will focus on compiling and mapping the most commonly used types of RTA criteria.

**Discussion:**

This systematic review will determine the most commonly used RTA criteria in adolescent patients post-ACL reconstruction. This will help future interventions build more effective adolescent-specific RTA assessments through the validation of current RTA criteria as well as the implementation of new criteria according to the identified literature gaps.

**Supplementary Information:**

The online version contains supplementary material available at 10.1186/s13643-022-01965-w.

## Background

Injuries to the anterior cruciate ligament (ACL) are increasing in prevalence in the adolescent population (10-18 years old) [1–3], with females aged 13-17 years possessing the highest injury incidence of any sex-age strata [4]. Following an ACL injury, a surgical reconstruction is typically pursued to restore knee stability and enable resumption of pre-injury activities [5]. However, only two-thirds of adolescent patients will return to their pre-injury levels of activity [6]. Furthermore, once an athlete has returned-to-sports, the risk for a subsequent ACL injury is considerably higher compared to the initial injury [7–9]. Approximately 17-19% of adolescent athletes will re-tear their ACL within two years following an ACL reconstruction [8, 10, 11], with greater than 30% of second ACL injuries occurring within the first 20 sport exposures following return-to-sports [8]. There is also a discrepancy between re-injury rates in adult and adolescent patients, with higher rates of second ACL injuries and revision surgeries in patients less than 18 years old, compared to older cohorts [9, 12].

A contributing factor to the high re-injury rates in the adolescent populations may be the lack of consensus regarding which criteria should be used when assessing readiness for unrestricted return-to-activity (RTA) [13]. RTA criteria typically refers to a set of tests, or test batteries, designed to incorporate a number of risk factors, the results of which can be used to clear athletes for RTA at the final stage of rehabilitation [14]. Despite the continuing development of milestone-based post-operative rehabilitation programs for young athletes [15], considerable debate remains regarding the optimal criteria for RTA clearance. Previous reviews have identified the most frequently used factors for determining RTA clearance following an ACL reconstruction [16], as well as the most commonly reported objective criteria [17]. Although these reviews have provided clinically meaningful findings, the studies focused primarily on an adult population, with no such evidence existing in adolescent patients. Considering the higher rates of re-injury in this population [8, 10, 11] and the identification of age-specific risk factors for ACL injury [18, 19], the treatment of ACL injuries in adolescent patients must be considered separately from adults. Notably, a recent scoping review provided an overview of the current evidence for RTA tests following an ACL reconstruction in adolescent patients; however, they did not identify what RTA tests are being used in clinical practice [20]. In addition, a recent survey of paediatric orthopaedic surgeons [13] and a review of children’s hospitals rehabilitation programs [21] found that the mode of testing and criteria thresholds for activity advancement varied considerably across hospitals and surgeons. Although these findings provide an estimate for the current landscape of surgeon practice [13], they may not accurately reflect RTA criteria used in scientific literature. By summarizing the scientific literature, future research can validate and adapt current RTA criteria, or target new areas for RTA development according to the identified literature gaps.

## Objective

The primary goal of this systematic review is to determine the criteria used when assessing RTA readiness post-ACL reconstruction in adolescent patients, as well as how commonly each criteria is used. For each article we will determine:

1. *How many RTA criteria were used?* Considering the psychological [22–24], biomechanical [25–29], and biological [30] changes that occur following an ACL reconstruction, it is likely that multiple metrics are required when assessing RTA readiness.
2. *Was the criteria time-based, subjective or objective?* Previous systematic reviews have shown that 42% of articles used time from ACL reconstruction as the only criterion when evaluating RTA [31]. This review will determine if these proportions are consistent in an adolescent population.
3. *What functional test or benchmark was met prior to RTA?* In order to validate current RTA criteria or target new areas for RTA development, the literature must be examined to determine the current standard-of-practice for RTA assessment.

We will also explore secondary outcomes, including determining the re-injury rate associated with each RTA assessment, as well as recording the most frequently used functional tasks and limb symmetry indexes (LSI).

## Methods

The Preferred Reporting Items for Systematic Reviews and Meta-Analyses (PRISMA) guidelines were followed when preparing this systematic review protocol (see Additional file 1 [32];). Any protocol modifications made during the conduct of the review will be described in the publication of the final report.

### Search strategy

An experienced university librarian assisted with the creation and execution of the search strategy (see Additional file 2). The search strategy draws upon existing search strings previously used in systematic reviews of ACL reconstruction RTA criteria [16, 17, 33]. Search terms will be entered under three concepts: concept 1 included terms “child,” “pediatric,” and “adolescent”; concept 2 included terms “anterior cruciate ligament reconstruction,” “ACL repair,” and “ACL surgery”; and concept 3 included terms “return to sport,” “return to play,” and “return to athletics.” Terms within each concept will be combined with the OR Boolean operator, and the three concepts will be combined with the AND Boolean operator. Where possible, terms will be mapped to medical subject headings and searched using keywords. The electronic databases MEDLINE, Embase, CINAHL, SPORTDiscus, and Cochrane Central Register of Controlled Trials will be searched from January 1, 2000, until submission of the final manuscript. The combination of these databases produces an estimated 97% recall of all primary studies involving orthopedic surgical interventions [34]. The search strategy will restrict citations to studies written in English and French. Although articles in other languages will be excluded, a list of the potentially relevant studies will be provided in a supplement of the final report for interested readers. To supplement the electronic database search, citations within all included studies will be manually reviewed to identify any additional studies omitted during the initial database searches.

### Study eligibility criteria

We set the eligibility criteria for the review according to the PICOS (population, intervention, comparison, outcomes, study design) framework [35]. We will include studies that meet the following criteria:

- *Population*: All adolescent patients who have undergone a primary ACL reconstructive surgery will be considered (10–18 years old at the time of surgery), without exclusions relative to patient sex or activity level*.*
- *Intervention*: A primary ACL reconstructive surgery*.* We will exclude articles where the patient is undergoing a revision ACL reconstruction. We will not restrict articles based on the graft type or surgical technique used.
- *Comparators*: Contralateral limb of patients with ACL reconstruction or patients unaffected by ACL rupture (healthy controls).
- *Outcomes*: We are interested in studies that specify the RTA criteria utilized following an ACL reconstruction. Studies will be excluded if they do not specify the criteria with enough detail to determine if the criteria were subjective or objective. From each articles, we will extract (i) how many criteria were used, (ii) the type of criteria (time-based, subjective, or objective), and (iii) the specific test or benchmark used.
- *Study design*: Study designs of interest will include observational studies (including cross-sectional studies and cohort studies) or randomized control trials. We will exclude conference proceedings, surgical techniques, technical notes, letters to editors, case reports, clinical commentaries, and review articles.

### Study selection

Publication details from all studies will be exported to Covidence systematic review software (Veritas Health Innovation, Melbourne, Australia; www.covidence.org), and duplicates will be removed. Study selection will be performed in two stages; screening at stage one will encompass reviewing titles and abstracts identified from the electronic searches. Two reviewers will independently review the title and abstract of each article identified through the literature search. All articles that meet the subject matter criteria described above will be included at this stage. Stage two screening will evaluate the full-text articles against the complete eligibility criteria, among those deemed potentially relevant during stage 1. Each article will be screened independently by two reviewers. Disagreements among reviewers will be decided through discussion, and a senior team member will be consulted if a disagreement can not be resolved. In addition, the authors of any studies with potential duplicate participants (e.g., same institution, overlapping patient enrollment dates) will be contacted to determine patient overlap. For articles with >50% patient overlap, the study with the larger patient population will be included [36]. Before each screening stage, we will calibrate the reviewers to ensure consistent application of eligibility criteria. We will continue the calibration until we reach ~95% agreement between the screeners. Finally, a PRISMA flow diagram will be prepared to document the study selection process in the final publication [32].

### Assessment of study quality

The quality of each study, including the risk of bias, will be assessed using the methodological index for non-randomized studies (MINORS) [37]. MINORS is a validated instrument developed because of the problems faced by clinicians given the lack of randomized surgical trials and the large number of observational studies in surgery [37]. The MINORS tool applies a scoring system across 12 items to assess the methodological and scientific value of studies, with the first 8 items relating to non-comparative studies and all 12 items relevant for comparative studies. The quality of each study will be independently assessed by two reviewers. Any disagreements will be resolved through discussion, with the involvement of a third reviewer if necessary. Articles will not be excluded on the basis of the assessment.

### Data extraction

A data extraction form will be developed and pilot tested using a sample of 5 articles and revised as necessary. One reviewer will extract the data, and two reviewers will verify the completeness of the extraction. Table 1 lists the items for data extraction. These items will constitute the elements of the standardized data extraction form used by reviewers.

**Table 1:** List of items for data extraction

*Study characteristics*	Author and date of publication
	Journal
	Study design
*Population characteristics*	Number of patients
	Patient’s sex
	Patient’s age at surgery
	Surgical technique and graft type
	Concomitant injuries
	Length of follow-up
*Outcomes of interest*	Criteria for RTA
	Proportion of patients who RTA
	Proportion of failed ACL reconstructions
	Proportion of contralateral ACL ruptures

### Data synthesis

Continuous variables will be recorded as the mean ± standard deviation (SD). If the mean or SD is not reported, it will be estimated according to a previously validated formula: (higher range value — lower range value)/4 or interquartile range/1.35 [38, 39]. Categorical variables (e.g., reinjury rate) will be recorded as frequencies with percentages. If identical RTA criteria are used for multiple cohorts within the same paper (e.g., male and female), then demographic (e.g., age range) will be combined and recorded together [40]. The primary outcome of interest was the RTA assessment used by each study when determining clinical clearance to full activities, recorded according to the following: (i) how many criteria were used; (ii) whether the criteria were time-based, subjective, or objective; and (iii) the specific test or benchmark used. As part of our secondary outcomes, we will record the reinjury rate associated with each RTA battery, as well as the most frequently used functional tasks.

## Discussion

The ACL is the most frequently damaged knee ligament [41], with rates continuing to rise among active adolescent athletes [1–3]. Despite surgical interventions aimed at restoring mechanical integrity [5], approximately 17–19% of adolescent athletes will sustain a second ACL injury within 2 years following an ACL reconstruction [8, 10, 11]. Given the high reinjury rate in this population [9, 12], and the potential for adverse long-term health consequences following an ACL injury [42–48], there is an urgent need to develop adolescent-specific RTA. This systematic review will identify the most commonly used criteria when determining unrestricted RTA following an adolescent ACL reconstruction. The results of this review will allow future interventions to build more effective adolescent-specific RTA assessments through the identification and validation of current RTA criteria and the implementation of new criteria according to the identified literature gaps.

A particular challenge for the present review will be the small number of studies conducted on adolescent ACL injuries. In anticipation of this, we made use of validated search strings developed in consultation with an experienced university librarian to maximize the coverage while retaining a feasible number of articles for screening. We have also included a secondary search of the included articles to identify any additional studies omitted during the initial database searches. Only studies which specify the adolescent-specific RTA criteria will be included in the final review. In addition, there may be variability in the descriptions of the utilized RTA criteria. Studies will only be included if they specified the RTA criteria with enough detail to determine if the criteria were subjective or objective. This will be independently assessed by two reviewers, with disagreement resolved through discussion. However, there is potential that some of the excluded investigations did in fact measure RTA criteria but did not include this information in the article. Finally, although the ACL reinjury rate will be extracted from each article, we may not be able to compare the ACL failure rates associated with specific RTA criteria. This type of analysis would require a separate investigation in which cohorts are carefully matched for graft type, sex ratio, chronicity of injury, concomitant injuries, articular cartilage deterioration, postoperative sports activity level, and time of follow-up. Therefore, future studies may be required to determine if the reported RTA criteria are effective in reducing ACL reinjury rates in an adolescent population.

We will publish the results of this review in a sports medicine research journal with the intent of maximizing outreach to healthcare professional and researchers pursuing research on ACL management. In addition to a peer-reviewed publication, we will also draft lay summaries to post online and for distribution to key societies, patient groups, and policymakers.

## Supplementary Information

**Additional file 1.** PRISMA-P 2015 Checklist.

**Additional file 2.** Search Algorithms.

## Data Availability

The datasets generated and/or analyzed during the current study will be available in the Open Science Foundation repository.

## Abbreviations

ACL: Anterior cruciate ligament
MINORS: Methodological index for non-randomized studies
PICOS: Population, intervention, comparison, outcomes, and study design
PRISMA: Preferred Reporting Items for Systematic Reviews and Meta-Analyses
RTA: Return to activity
SD: Standard deviation
