# Supplementary material for: Protocol for the systematic review of return-to-activity criteria in adolescent patients following an anterior cruciate ligament reconstruction
Source: Syst Rev. 2022 May 14;11:93. doi: 10.1186/s13643-022-01965-w (PMC9107732; doi:10.1186/s13643-022-01965-w)
Supplement: Supplementary file 2 — Additional file 2. Search Algorithms. [file 13643_2022_1965_MOESM2_ESM.docx]

| Search Algorithms | | | | | |
| --- | --- | --- | --- | --- | --- |
|  | **MEDLINE - OVID** | **EMBASE - OVID** | **COCHRANE RCT - OVID** | **CINAHL - EBSCO** | **SPORTDiscus - EBSCO** |
| Concept 1  Subject Headings | *Child/*  *Adolescent/*  *Pediatrics/* | *Child/*  *Adolescent/*  *Pediatrics/* | *Child/*  *Adolescent/*  *Pediatrics/* | MH child OR  MH adolescence OR MH Pediatrics | SU Children OR  SU Teenagers OR  SU Pediatrics |
| Concept 1  Key Word Search | *((high or secondary or middle or elementary) adj2 school*).ti,ab*  *(skeletal* adj3 immatur*).ti,ab.*  *(child* or young* or juvenile* or kid* or adolescen* or teen* or youth* or p?ediatric* or prepubescent*).ti,ab.* | *((high or secondary or middle or elementary) adj2 school*).ti,ab*  *(skeletal* adj3 immatur*).ti,ab.*  *(child* or young* or juvenile* or kid* or adolescen* or teen* or youth* or p?ediatric* or prepubescent*).ti,ab.* | *((high or secondary or middle or elementary) adj2 school*).ti,ab*  *(skeletal* adj3 immatur*).ti,ab.*  *(child* or young* or juvenile* or kid* or adolescen* or teen* or youth* or p?ediatric* or prepubescent*).ti,ab.* | TI (((high or secondary or middle or elementary) N2 school*) ) OR  AB ( ((high or secondary or middle or elementary) N2 school*) )  TI (skeletal* N3 immatur*) OR  AB (skeletal* N3 immatur*)  TI ( (child* or young* or juvenile* or kid* or adolescen* or teen* or youth* or p#ediatric* or prepubescent*) ) OR AB ( (child* or young* or juvenile* or kid* or adolescen* or teen* or youth* or p#ediatric* or prepubescent*) ) | TI ( (high or secondary or middle or elementary) N2 school*) ) OR  AB ( (high or secondary or middle or elementary) N2 school*) )  TI (skeletal* N3 immatur*) OR  AB (skeletal* N3 immatur*)  TI ( (child* or young* or juvenile* or kid* or adolescen* or teen* or youth* or p#ediatric* or prepubescent*) ) OR AB ( (child* or young* or juvenile* or kid* or adolescen* or teen* or youth* or p#ediatric* or prepubescent*) ) |
| Concept 1  (final) | 1 or 2 or 3 or 4 | 1 or 2 or 3 or 4 | 1 or 2 or 3 or 4 | S1 or S2 or S3 or S4 | S1 or S2 or S3 or S4 |
|  |  |  |  |  |  |
| Concept 2 Subject Headings | exp Anterior Cruciate Ligament Reconstruction/ | exp Anterior Cruciate Ligament Reconstruction/ | exp Anterior Cruciate Ligament Reconstruction/ | MH anterior cruciate ligament reconstruction | SU anterior cruciate ligament surgery |
| Concept 2  Key Word Search | ((ACL or anterior cruciate ligament) adj4 (reconstruction* or repair* or graft* or surger* or operat*)).ti,ab. | ((ACL or anterior cruciate ligament) adj4 (reconstruction* or repair* or graft* or surger* or operat*)).ti,ab. | ((ACL or anterior cruciate ligament) adj4 (reconstruction* or repair* or graft* or surger* or operat*)).ti,ab. | TI ( ((ACL or anterior cruciate ligament) N4 (reconstruction* or repair* or graft* or surger* or operat*)) ) OR  AB ( ((ACL or anterior cruciate ligament) N4 (reconstruction* or repair* or graft* or surger* or operat*)) ) | TI ( ((ACL or anterior cruciate ligament) N4 (reconstruction* or repair* or graft* or surger* or operat*)) ) OR  AB ( ((ACL or anterior cruciate ligament) N4 (reconstruction* or repair* or graft* or surger* or operat*)) ) |
| Concept 2 (final) | 6 or 7 | 6 or 7 | 6 or 7 | S6 or S7 |  |
|  |  |  |  |  |  |
| Concept 3  Subject Headings | return to sport/ | return to sport/ | "Recovery of Function"/ | MH Sports Re-Entry | SU Sport Participation |
| Concept 3  Key Word Search | (return* to adj3 (sport* or play* or activit* or athletic* or participation*)).ti,ab. | (return* to adj3 (sport* or play* or activit* or athletic* or participation*)).ti,ab. | (return* to adj3 (sport* or play* or activit* or athletic* or participation*)).ti,ab. | TI ( ("return* to" N3 (sport* or play* or activit* or athletic* or participation*)) ) OR  AB ( ("return* to" N3 (sport* or play* or activit* or athletic* or participation*)) ) | TI ( ("return* to" N3 (sport* or play* or activit* or athletic* or participation*)) ) OR AB ( ("return* to" N3 (sport* or play* or activit* or athletic* or participation*)) ) |
| Concept 3 (Final) | 9 or 10 | 9 or 10 | 9 or 10 | S9 or S10 | S9 or S10 |
|  |  |  |  |  |  |
| Language | English or French | English or French | English or French | English or French | English or French |
| Years | 2000-2021 | 2000-2021 | 2000-2021 | 2000-2021 | 2000-2021 |
| Final search | 5 and 8 and 11 | 5 and 8 and 11 | 5 and 8 and 11 | S5 AND S8 AND S11 |  |
|  |  |  |  |  |  |
| *Date Search Executed* | N/A | N/A | N/A | N/A | N/A |
